# Synthesis and pH-responsive dissociation of framboidal ABC triblock copolymer vesicles in aqueous solution†
†Electronic supplementary information (ESI) available: Full experimental details for the synthesis and characterisation of the diblock and triblock copolymer vesicles, assigned ^1^H NMR spectra, GPC traces, digital images of dispersions, additional plots of pH *vs.* hydrodynamic diameter, count rate and zeta potential, a table of SAXS parameters, extra data for G_58_H_250_D_184_ and un-merged SAXS patterns are provided. See DOI: 10.1039/c7sc04788f


**DOI:** 10.1039/c7sc04788f

**Published:** 2017-12-13

**Authors:** C. J. Mable, L. A. Fielding, M. J. Derry, O. O. Mykhaylyk, P. Chambon, S. P. Armes

**Affiliations:** a Department of Chemistry , Uni. of Sheffield , Dainton Building, Brook Hill , Sheffield , South Yorkshire S3 7HF , UK . Email: S.P.Armes@sheffield.ac.uk ; Tel: +44 (0)114 222 9342; b Department of Chemistry , Uni. of Liverpool , Crown Street , Liverpool , L69 7ZD , UK; c School of Materials , Uni. of Manchester , Oxford Rd , Manchester , M13 9PL , UK

## Abstract

A series of pH-responsive all-methacrylic ABC triblock copolymer vesicles were prepared from precursor diblock copolymer vesicles *via* RAFT seeded emulsion polymerisation. Microphase separation between the two hydrophobic membrane-forming B and C blocks produced a distinctive framboidal morphology, for which the mean globule size can be tuned by adjusting the triblock copolymer composition. These vesicles remain intact at neutral pH, but undergo irreversible dissociation on addition of acid as a result of protonation of the tertiary amine groups located within the third block. Small-angle X-ray scattering (SAXS) was utilised to characterise the morphologies formed at pH 8 and pH 3. According to time-resolved SAXS studies, the acid-induced dissociation of these pH-responsive framboidal vesicles involves appreciable membrane swelling within 50 ms and is complete.

## Abstract 2

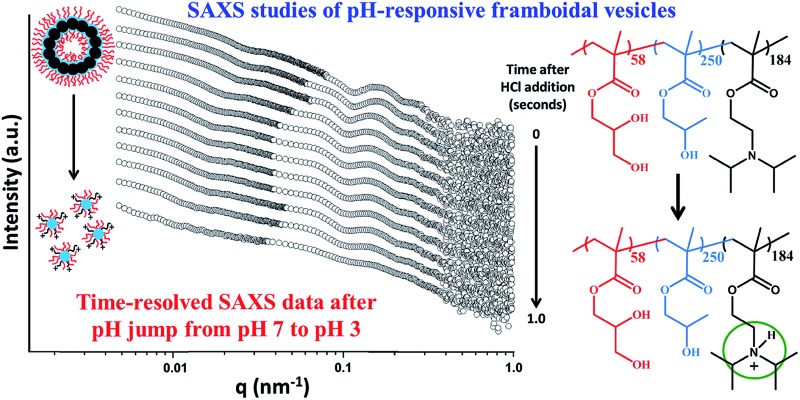
A series of framboidal ABC triblock copolymer vesicles were prepared from precursor diblock copolymer vesicles *via* RAFT seeded emulsion polymerisation and their acid-induced dissociation was characterised by SAXS.

## Introduction

It is well-known that amphiphilic block copolymers undergo spontaneous self-assembly in aqueous solution.^1^–^4^ For example, many variations on the vesicular morphology have been reported in the literature, including stomatocyte-like,^5^ Janus-type^6^ and framboidal vesicles.^7^–^9^ Recent advances in polymerisation-induced self-assembly (PISA)^10^–^12^ have enabled a wide range of block copolymer nanoparticles to be prepared directly in concentrated aqueous solution *via* reversible addition-fragmentation chain transfer (RAFT) aqueous dispersion polymerisation.^13^,^14^ Such one-pot syntheses are much more convenient than traditional post-polymerisation processing techniques, which typically involve a solvent or pH switch^15^–^18^ or thin film rehydration,^19^,^20^ and are invariably restricted to dilute solution. For example, a poly(glycerol monomethacrylate) macromolecular chain transfer agent (PGMA macro-CTA) can be chain-extended using 2-hydroxypropyl methacrylate (HPMA).^14^,^21^,^22^ Self-assembly occurs to form PHPMA-core nanoparticles stabilised by the water-soluble PGMA chains. Depending on the precise formulation, a range of copolymer morphologies can be achieved, including spheres, worms and vesicles.^21^–^23^ Recently, Chambon *et al.* utilised RAFT seeded emulsion polymerisation to grow a third water-insoluble-monomer (benzyl methacrylate) from PGMA–PPMA diblock copolymer vesicles. This leads to microphase separation within the vesicle membrane, resulting in the formation of ABC triblock copolymer vesicles with a distinctive framboidal (*i.e.* raspberry-like) morphology.^7^ Such model colloids were used to study the effect of varying the surface roughness on Pickering emulsifier performance.^9^

There are many PISA syntheses of thermoresponsive nano-objects in the literature.^11^,^13^,^24^–^32^ However, there are rather fewer reports of pH-responsive nanoparticles prepared *via* PISA.^8^,^27^,^33^–^37^ Moreover, as far as we are aware, there is only one other literature report of pH-responsive framboidal nanoparticles: such phenylboronic acid-functionalised nano-objects were prepared by Hasegawa *et al. via* aqueous dispersion polymerisation using conventional free radical chemistry.^8^

**Fig. 1:**
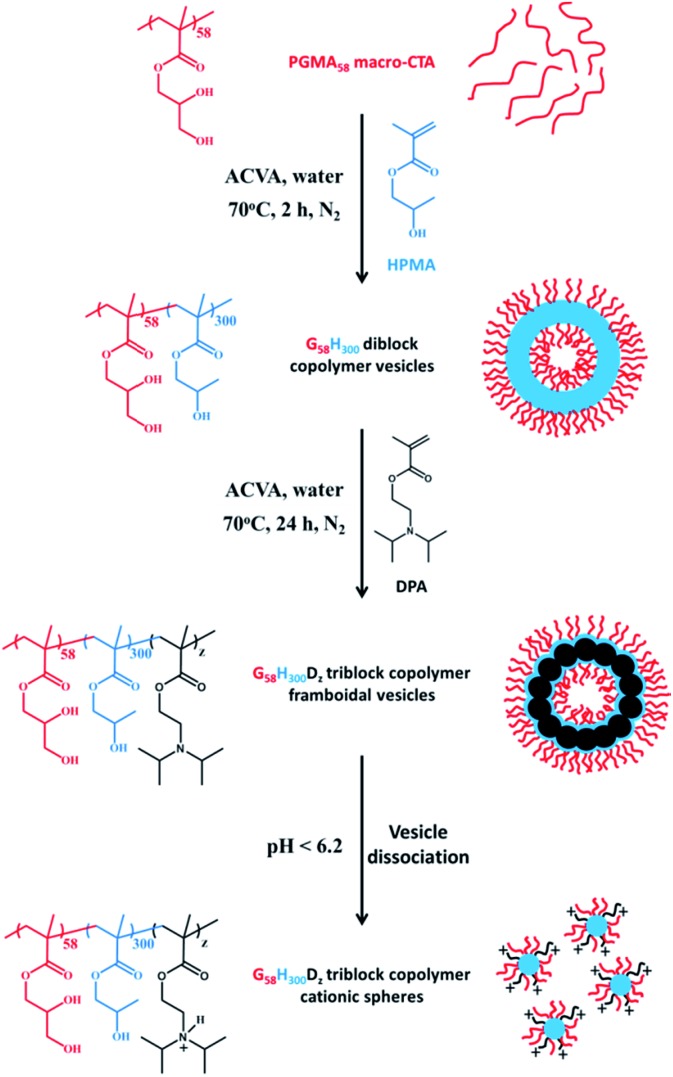
Synthetic route to 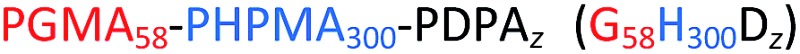 framboidal triblock copolymer vesicles *via* polymerisation-induced self-assembly (PISA) in aqueous solution at pH 7–8. Addition of acid causes protonation of the tertiary amine residues, which renders the PDPA block hydrophilic and induces vesicle dissociation. Inset: schematic cartoon of precursor vesicles, framboidal vesicles and cationic spheres.

Herein we report the chain extension of PGMA–PHPMA diblock copolymer vesicles using varying amounts of 2-(diisopropylamino)ethyl methacrylate (DPA) to produce novel framboidal PGMA–PHPMA–PDPA triblock copolymer vesicles, see Fig. 1. For brevity, such copolymers are hereafter denoted as G_58_H_300_D_*z*_, where the subscripts refer to the mean degree of polymerisation, DP, of each block and *z* is a variable.

## Results and discussion

Initially, a PGMA_58_ macro-CTA was chain-extended *via* RAFT aqueous dispersion polymerisation of HPMA at 15% w/w solids to prepare a 100 gram batch of G_58_H_300_ diblock copolymer vesicles, see Fig. 1. These G_58_H_300_ precursor vesicles were then diluted to 10% w/w and subsequently chain-extended using varying amounts of DPA *via* RAFT seeded emulsion polymerisation to prepare a series of G_58_H_300_D_*z*_ triblock copolymers, where *z* was adjusted from 86 to 460 (allowing for incomplete reaction of the DPA monomer). ^1^H NMR studies indicated that more than 99% HPMA conversion was achieved within 2 h at 70 °C (as expected based on previous work^21^), while DPA conversions of 82–92% were achieved within 24 h at 70 °C. ^1^H NMR studies of the final G_58_H_300_D_*z*_ triblock copolymers were conducted in CD_3_OD containing 4% DCl, which is a good solvent for all three blocks (see Fig. S1 in ESI† for assigned spectra).

DMF GPC studies conducted using a series of poly(methyl methacrylate) (PMMA) calibration standards indicated that a near-monodisperse G_58_H_300_ diblock copolymer precursor (*M*_w_/*M*_n_ = 1.12; see Fig. S2 in ESI†) was obtained with a relatively high blocking efficiency. This GPC trace had a weak high molecular weight shoulder, which was attributed to light branching caused by small amounts of dimethacrylate impurity within the HPMA monomer (0.07 mol% as judged by HPLC). In the case of the G_58_H_300_D_*z*_ triblock copolymers, the PDPA block is DMF-insoluble, while the PGMA block is THF-insoluble, rendering GPC characterisation of this triblock copolymer rather problematic. To render these triblock copolymers THF-soluble for GPC analysis, the PGMA block was derivatised according to a previously reported protocol.^38^,^39^ Thus, such G_58_H_300_D_*z*_ triblock copolymers were dissolved in pyridine, excess benzoic anhydride (four equivalents based on GMA and HPMA residues) was added, and esterification of the hydroxyl groups was allowed to proceed for 24 h at room temperature. The benzoate-protected copolymers obtained by this method were always fully soluble in THF (unlike their precursors). The GPC traces were unimodal and indicated high blocking efficiencies (see Fig. S3 in ESI†). However, dispersities were greater than that expected for RAFT polymerisations (see Table 1). To determine whether this observation was real or merely a GPC artefact, the PGMA_58_ macro-CTA and G_58_H_300_ diblock copolymer precursor were also derivatised so that the DMF GPC and THF GPC protocols could be directly compared (Table 1). Similar number-average molecular weight (*M*_n_) values were obtained for the original homopolymer *via* DMF GPC and the esterified copolymer *via* THF GPC, as expected. However, the dispersities are significantly higher for the latter (see Table 1). Thus the dispersities obtained for G_58_H_300_D_*z*_ triblock copolymers where *z* is 86, 164 or 249 are likely to be an artefact of the esterification protocol. However, the much higher dispersities (*M*_w_/*M*_n_ > 3.0) obtained when *z* = 356 or 460 are perhaps less likely to be solely owing to such an artefact. For these latter two copolymers, the RAFT polymerisation of the DPA was clearly not well-controlled, giving rise to relatively broad molecular weight distributions. Similar results have been recently reported by Derry *et al.* for other PISA formulations when targeting relatively high degrees of polymerisation.^40^

**Table 1:** Summary of ^1^H NMR calculated composition, GPC number-average molecular weight (*M*_n_) and dispersity (*M*_w_/*M*_n_), DLS hydrodynamic diameter (*D*_h_), derived count rate and zeta potential, and SAXS-derived globule diameter, membrane thickness and vesicle diameter obtained for framboidal G_58_H_300_D_*z*_ triblock copolymer vesicles (where the mean degree of polymerisation of the third block, *z*, ranges from 86 to 460). Relevant data for the corresponding G_58_ macro-CTA and linear G_58_H_300_ diblock copolymer precursor vesicles are included for comparison

Copolymer composition	*M*_n_ (g mol^–1^)	*M*_w_/*M*_n_	*D*_h_ (PDI)/nm		DLS count rate/kcps		Zeta potential/mV		Globule diameter/nm	Membrane thickness/nm	Vesicle diameter^*c*^/nm
			pH 8	pH 3	pH 8	pH 3	pH 8	pH 3			
G_58_ macro-CTA	15 400^*a*^, 14 500^*b*^	1.13^*a*^, 1.36^*b*^									
G_58_H_300_	66 700^*a*^, 62 600^*b*^	1.12^*a*^, 1.41^*b*^	396 (0.08)	388 (0.09)	55 200	49 000	–11	–2.0		16.8	383
G_58_H_300_D_86_	71 900^*b*^	1.67^*b*^	372 (0.11)	44 (0.42)	47 900	800	–15	+34	27	24.9	391
G_58_H_300_D_164_	76 600^*b*^	1.98^*b*^	379 (0.12)	67 (0.58)	46 300	700	–19	+36	37	36.3	402
G_58_H_300_D_249_	84 800^*b*^	1.98^*b*^	403 (0.07)	122 (0.49)	42 100	1100	–28	+37	44	46.4	412
G_58_H_300_D_356_	91 600	3.08^*b*^	409 (0.13)	293 (0.63)	28 600	1600	–31	+40			
G_58_H_300_D_460_	119 800^*b*^	3.55^*b*^	442 (0.15)	1413 (0.65)	19 600	2600	–39	+41			

^*a*^Data obtained *via* DMF GPC (against PMMA standards) using a refractive index detector.

^*b*^Data obtained *via* THF GPC (against PMMA standards) using a refractive index detector after exhaustive esterification of the hydroxyl groups.

^*c*^These data are only considered to be approximate, because the SAXS camera length was not long enough to enable accurate calculation of the overall vesicle diameters.

**Fig. 2:**
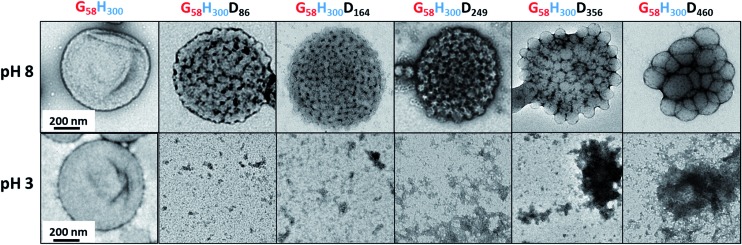
Representative TEM images obtained for the G_58_H_300_ precursor vesicles and a series of G_58_H_300_D_*z*_ framboidal vesicles, where *z* = 86, 164, 249, 356 or 460 (from left to right) at pH 8 (top) and pH 3 (bottom). The scale bars shown in the far left-hand images are correct for all images shown at that particular pH.

TEM studies of the G_58_H_300_ diblock copolymer revealed a pure vesicular morphology (see Fig. 2). As expected, these precursor vesicles were not pH-responsive: they remained intact at both pH 8 and pH 3. TEM studies of the G_58_H_300_D_*z*_ triblock copolymers confirmed the formation of framboidal vesicles at pH 8. This distinctive morphology is the result of microphase separation between the PHPMA and PDPA membrane-forming blocks, which becomes more pronounced with increasing *z*. Unlike the diblock precursor vesicles, these framboidal triblock copolymer vesicles do not remain intact at pH 3: disintegration is observed by TEM (see Fig. 2). Visual inspection of the dispersion is also consistent with loss of the vesicular morphology. At pH 8, the vesicle dispersions are highly turbid as expected, but at pH 3 a relatively clear solution is obtained. When returning to pH 8, a white precipitate is formed, indicating that this pH-responsive behaviour is not reversible, presumably because the framboidal morphology is kinetically-trapped (see Fig. S4 in ESI†).

Dynamic light scattering (DLS) measurements indicate that vesicle dissolution occurs when the pH is lowered from pH 8 to pH 3 (see Table 1). At pH 8, the G_58_H_300_ diblock copolymer vesicles have a hydrodynamic diameter of ∼396 nm. Chain extension with 86 DPA units led initially to a modest reduction in the mean vesicle diameter to 372 nm. Presumably, this initial compaction is the result of a more hydrophobic membrane. For higher *z* values, the hydrodynamic diameter gradually increases up to 442 nm for G_58_H_300_D_460_. This reflects formation of progressively thicker membranes, with the concomitant evolution of the distinctive framboidal morphology.

**Fig. 3:**
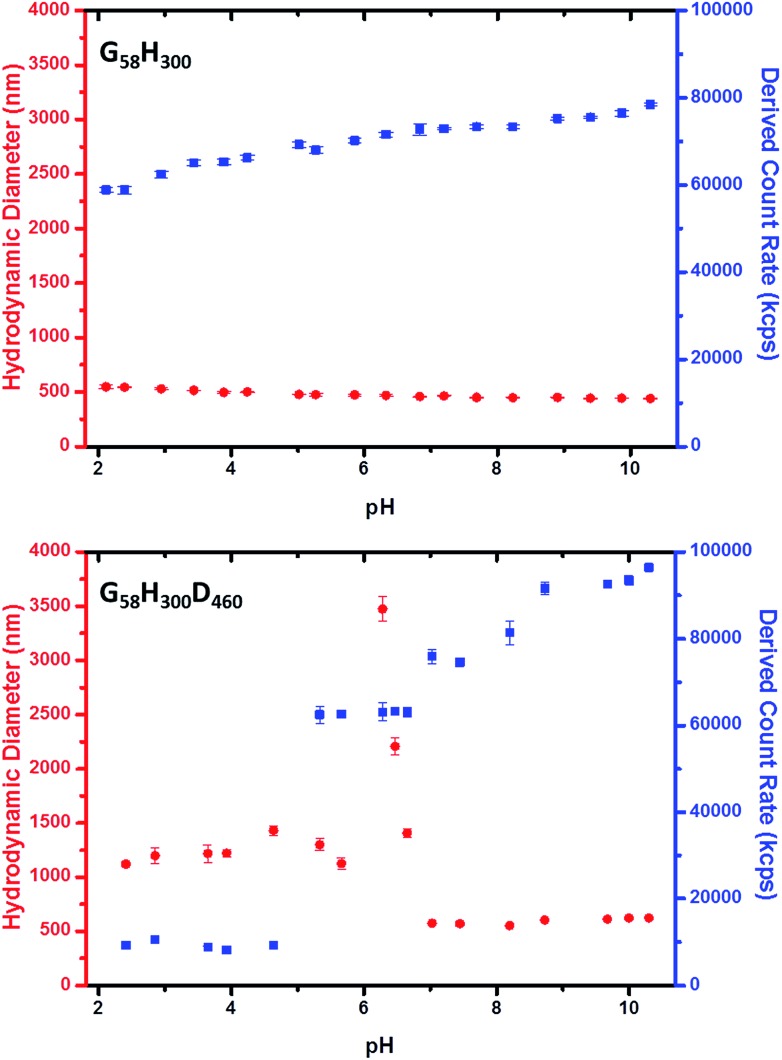
Variation in hydrodynamic diameter (
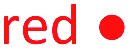
) and count rate (
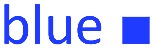
) with solution pH for (top) G_58_H_300_ diblock copolymer precursor vesicles and (bottom) G_58_H_300_D_460_ triblock copolymer framboidal vesicles.

At pH 3, the G_58_H_300_ precursor vesicles have a comparable hydrodynamic diameter and polydispersity to that observed at pH 8. In contrast, the series of G_58_H_300_D_*z*_ triblock copolymer vesicles typically exhibit significantly smaller hydrodynamic diameters and higher polydispersities at pH 3 compared to those observed at pH 8. The exception is G_58_H_300_D_460_, for which a relatively large hydrodynamic diameter of 1413 nm is observed. DLS studies were also conducted as a function of pH in the presence of 1 mM KCl (see Fig. 3 and S5 in the ESI†). The G_58_H_300_ precursor vesicles maintained a constant hydrodynamic diameter of around 400–500 nm over a wide range of pH, while the scattered light intensity (or count rate) is only reduced by around 20% in acidic solution (see Fig. 3). In contrast, the count rate observed for the G_58_H_300_D_460_ triblock copolymer vesicles is reduced by approximately an order of magnitude (from ∼10^5^ kcps at pH 9 to ∼10^4^ kcps at pH 4), with a concomitant increase in the apparent hydrodynamic diameter from 500 nm up to 1250 nm.

Close inspection of the DLS data suggests that disruption of the framboidal vesicle morphology occurs when the pH is lowered below the p*K*_a_ of the PDPA block, which is approximately 6.2 (see Fig. 3). At around this p*K*_a_ value, the hydrophobic PDPA chains within the vesicle membrane gradually become protonated and therefore acquire cationic character (see Fig. 1). The partially protonated vesicles pass through their isoelectric point (IEP) at around pH 6, which induces flocculation. Below the IEP, partial vesicle disintegration occurs and relatively loose, weakly-interacting colloidal aggregates of rather ill-defined morphology are formed, as judged by TEM and DLS studies (see Fig. 2 and 3). It is emphasised that molecularly-dissolved triblock copolymer chains are not produced at low pH, because the relatively long central PHPMA block retains its weakly hydrophobic character under these conditions.

**Fig. 4:**
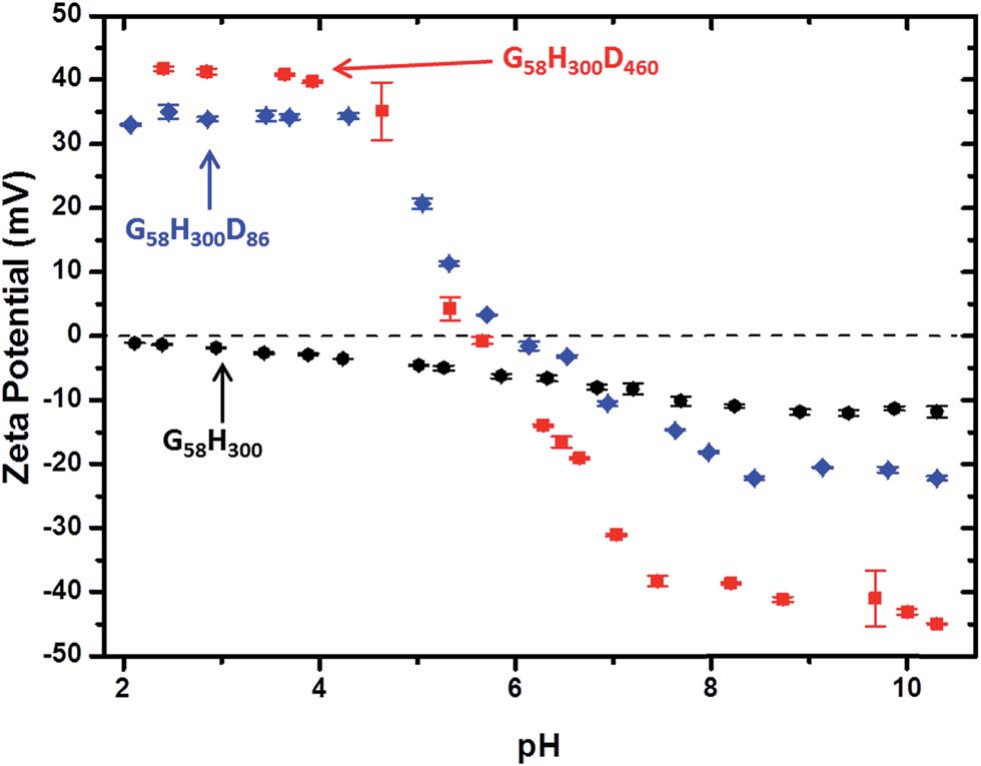
Zeta potential *vs.* pH curves obtained for: (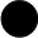) G_58_H_300_ diblock copolymer precursor vesicles, (
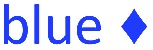
) G_58_H_300_D_86_ triblock copolymer vesicles and (
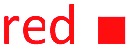
) G_58_H_300_D_460_ triblock copolymer vesicles.

Aqueous electrophoresis studies were conducted on the G_58_H_300_ precursor vesicles and both the G_58_H_300_D_86_ and G_58_H_300_D_460_ framboidal vesicles as a function of pH (see Fig. 4). The latter two samples exhibited isoelectric points at around pH 6, with the positive zeta potentials of +34 to +42 mV observed at low pH being attributed to protonation of the tertiary amine groups located within in the PDPA block. This is consistent with the observation of a dramatic reduction in aggregate size in each case, as judged by TEM (see Fig. 2) and DLS (see Fig. 3 and S4 in ESI†).

In contrast, the diblock copolymer precursor vesicles did not exhibit any IEP, as expected. Above around pH 6, all three types of vesicles remained intact and exhibited anionic character, possibly owing to selective adsorption of hydroxide ions onto the vesicle surface.^41^ Aqueous electrophoresis studies were also conducted on the other copolymers (see Fig. S6 in ESI†). Considering the TEM, DLS and zeta potential data together, three distinct physical states for these G_58_H_300_D_*z*_ triblock copolymer particles can be identified. Small cationic copolymer aggregates are formed below pH 5, weakly cationic copolymer vesicles are obtained at pH 5–6, and anionic framboidal vesicles are produced above pH 6.2.

**Fig. 5:**
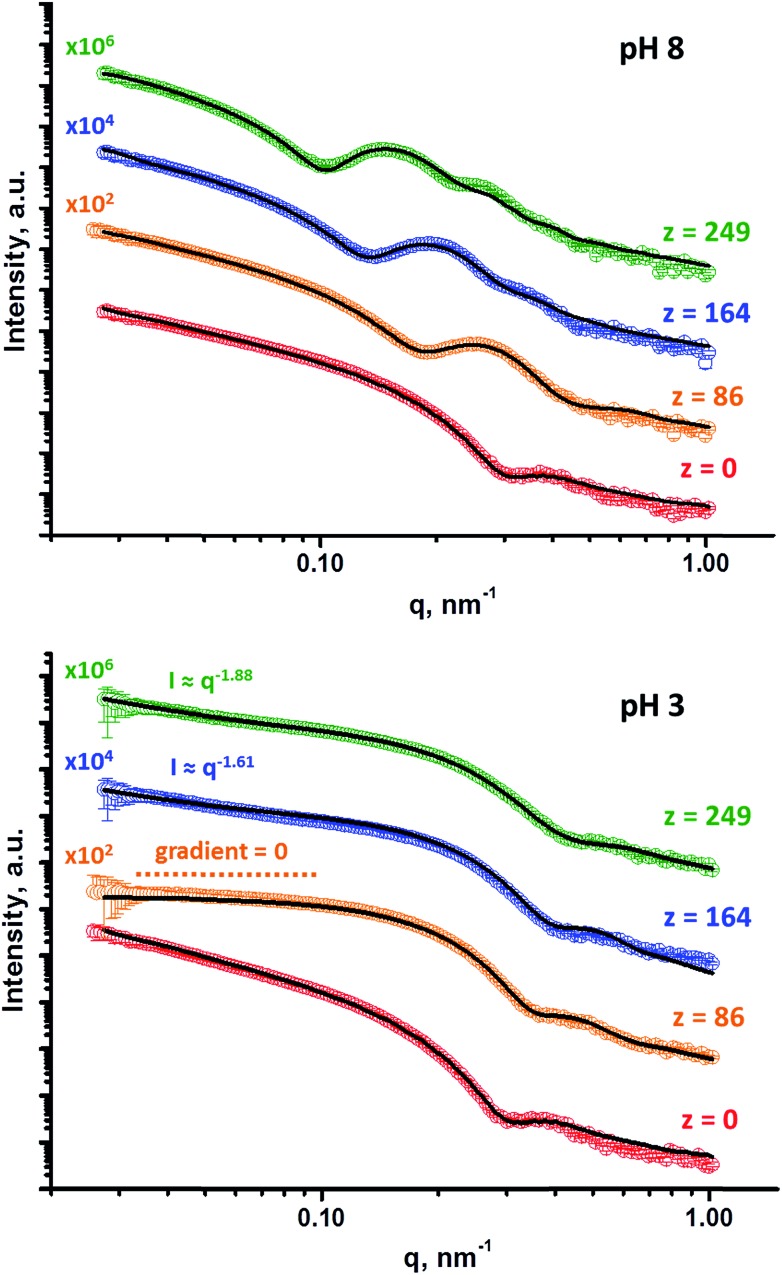
SAXS patterns obtained at pH 8 and pH 3 for 1.0% w/w aqueous dispersions of G_58_H_300_ diblock copolymer precursor vesicles (where *z* = 0) and a series of framboidal G_58_H_300_D_*z*_ triblock copolymer vesicles, where *z* = 86, 164, 125, 249. Solid lines represent fitting curves. At pH 8, a single vesicle model was sufficient for *z* = 0, whereas the superposition of a vesicle model and a spherical micelle model were required for higher *z* values. At pH 3, a single vesicle model was sufficient for *z* = 0 and a single spherical micelle model was sufficient for *z* = 86, whereas superposition of a spherical micelle and a mass fractal model was required for higher *z* values. For clarity, these SAXS patterns are shifted upward by an arbitrary factor, as indicated in the figure.

SAXS analyses (see Fig. 5) were conducted to obtain reliable framboidal vesicle globule dimensions and also to provide further insight with regard to the copolymer morphology formed at pH 3. TEM images indicate that the G_58_H_300_ precursor vesicles have smooth membranes. In contrast, the highly distinctive framboidal morphology possessed by the G_58_H_300_D_*z*_ triblock copolymer vesicles dried at pH 8 is comparable to that of the polymer core-particulate silica shell particles reported by Balmer and co-workers.^42^–^45^ In this earlier work, Monte Carlo simulations were utilised to demonstrate^42^ that the SAXS patterns obtained for such nanocomposite particles could be described by a two-population model. This model is represented by a superposition of two scattering signals originating from a core–shell structure comprising a spherical latex core surrounded by a shell composed of small spherical silica nanoparticles (population 1) and the many silica nanoparticles that form this shell (population 2).

A similar approach was recently adopted by Mable *et al.* for the SAXS analysis of closely-related pH-invariant framboidal triblock copolymer vesicles.^9^ In this more recent study, population 1 represented the vesicles and population 2 described the micelle-like spherical globules formed within the vesicle membrane. Herein, we use the same two-population SAXS model for fitting the G_58_H_300_D_*z*_ patterns recorded at pH 8.

Population 1 corresponds to smooth vesicles and is thus also appropriate for SAXS analysis of the G_58_H_300_ diblock copolymer precursor. This approach^46^ produced a reasonably good fit to the SAXS pattern over six orders of magnitude of X-ray scattering intensity (Fig. 5, pH 8, red data). The volume-average vesicle diameter was estimated to be 383 nm (unfortunately, the camera length used to collect these SAXS data was not long enough to provide a more reliable value for this parameter). Nevertheless, this is consistent with both TEM observations (Fig. 2) and DLS data (Table 1). According to TEM analysis, the mean vesicle diameter was about 350 nm, while DLS studies indicated a mean hydrodynamic vesicle diameter (*D*_h_) of 396 nm with a polydispersity index (PDI) of 0.08. The radius of gyration (*R*_g_) of the G_58_ corona block was determined to be 2.4 nm from model fitting of the G_58_H_300_ SAXS pattern. This experimental value is comparable to a theoretical estimate: the projected contour length of a single GMA monomer is 0.255 nm (two carbon bonds in all-trans conformation), the total contour length of a G_58_ block, *L*_PGMA_ = 58 × 0.255 nm = 14.79 nm and the Kuhn length of 1.53 nm, based on the literature value for poly(methyl methacrylate),^47^ result in an estimated *R*_g_ of (14.79 × 1.53/6)^1/2^, or 1.94 nm. The water volume fraction, *x*_sol_, within the membrane is approximately 0.50 according to the SAXS data fit. This is relatively high and reflects the weakly hydrophobic nature of the PHPMA block.^24^ As expected, when acid is added to the aqueous dispersion of G_58_H_300_ diblock copolymer vesicles to lower its solution pH, these vesicles remain unchanged because they are not pH-responsive (see Fig. 5, pH 3, red data).

Superposition of scattering signals from populations 1 and 2 (corresponding to vesicles and spherical micelles, respectively),^9^,^48^ was essential to produce satisfactory fits to the SAXS patterns obtained for the G_58_H_300_D_*z*_ framboidal vesicles, where *z* is 86, 164 or 249 (Fig. 5, pH 8). Previously, we assumed that both the *R*_g_ of the PGMA block and the water content within the hydrophobic part of the vesicle membrane remained constant, regardless of whether closely-related triblock copolymer vesicles were smooth or framboidal.^9^ A similar assumption was made in the current study, and the *R*_g_ and *x*_sol_ values determined for the G_58_H_300_ precursor vesicles were fixed when fitting the SAXS patterns recorded for the G_58_H_300_D_*z*_ framboidal vesicles. This self-consistent analytical approach indicated that the thickness of the hydrophobic component of the vesicle membrane (*T*_mc_) increased when targeting higher DPs for the PDPA block (see Table S1 in the ESI†). In addition, both TEM observations (Fig. 2) and DLS studies (Table 1) suggest that the vesicle diameter remained virtually constant over all copolymer compositions. Taken together, these data suggest that the vesicle growth mechanism involves a gradual reduction in the vesicle lumen volume, as reported by Warren and co-workers where non-framboidal G_55_H_*y*_ vesicles, for *y* = 200 to 2000.^49^ Similar observations were reported by Mable *et al.* when chain-extending G_63_H_350_ precursor vesicles with benzyl methacrylate (B) to obtain G_63_H_350_B_*z*_ framboidal vesicles, where *z* ranged from 25 to 400.^9^ The nanoscale phase separation that occurs within the vesicle membrane described by the spherical micelle model (population 2) can be verified by SAXS analysis. Both the spherical micelle radius (*R*_s_) and the relative concentration of the second population (*c*_2_/*c*_1_) increase when targeting higher PDPA block volume fractions, *V*_PDPA_, (see Table S1 in the ESI†). SAXS analysis indicates that the mean micelle/globule diameter (*D*_s_ = 2*R*_s_ + 4*R*_g_) for the G_58_H_300_D_*z*_ framboidal vesicles increases from 27 nm to 44 nm as *z* is varied from 86 to 249.

Nevertheless, some deviations between the fitting pattern and the experimental pattern are discernible for G_58_H_300_D_249_. The pronounced feature observed in the experimental SAXS pattern at *q* ∼ 0.25 nm^–1^ cannot be fully reproduced by the model fit. In principle, growth of the PDPA block within the hydrophobic membrane may drive its strong segregation from the PHPMA block, producing two regions of differing electron density within the spherical globule cores. If this explanation is correct, the inner core is likely to be the highly hydrophobic PDPA block while the outer core should contain the weakly hydrophobic PHPMA block. To examine this hypothesis, the two-population model was further refined. For population 1, the hydrophobic component of the vesicle membrane was assumed to comprise an inner PDPA layer surrounded by two outer PHPMA layers. Similarly, the hydrophobic spherical micelle core associated with population 2 was assumed to have a core–shell structure, whereby the core contained the PDPA blocks and the PHPMA blocks were located within the shell. However, this more sophisticated model did not produce an improved data fit compared to the original two-population model. This suggests that the deviation observed between the experimental and fitting patterns is not related to strong segregation between these two hydrophobic blocks. It is perhaps worth emphasising here that the two-population model describes the vesicles and spherical micelles independently: it does not include cross terms between these two structural features. In the literature, attempts have been made to account for such cross terms for other complex multicomponent particles.^50^ However, various additional parameters such as vesicle and spherical micelle polydispersities are required for such models. This significantly complicates the analysis and is considered to be beyond the scope of the current study.

It was also difficult to obtain satisfactory fits to the SAXS patterns obtained for G_58_H_300_D_356_ and G_58_H_300_D_460_ framboidal vesicles using the basic two-population model. As recently reported in the literature, the vesicle growth mechanism during PISA leads to a gradual reduction in the volume of the vesicle lumen.^40^ Because these two vesicles possess relatively long PDPA blocks, the vesicle membrane is rather thick, resulting in a substantially reduced vesicle lumen volume. Moreover, these framboidal vesicles can no longer be described as spherical globules located on a vesicle surface. Instead, the micelle-like globules become so large that these nanoparticles are essentially a cluster (aggregate) of pseudo-spherical globules. For example, the *c*_2_/*c*_1_ ratio increases from 0.273 to 0.997 as *z* is varied from 86 to 249. Thus, the relative concentration of micelle-like globules is essentially the same as that of the vesicles when *z* = 249. For higher PDPA block DPs (*e.g. z* = 356 or 460), the *c*_2_/*c*_1_ ratio increases further, indicating a significant reduction in the relative vesicle concentration. Similar findings were reported recently by Mable *et al.* when analysing G_63_H_350_B_z_ framboidal vesicles. The two-population model did not provide a satisfactory fit to the SAXS pattern recorded for vesicles prepared when targeting the longest PBzMA block DP (*z* = 400), most likely for the same reason.^9^ Instead, an alternative ‘aggregated sphere’ model should be used to obtain satisfactory SAXS data fits. Given this literature precedent, the two G_58_H_300_D_*z*_ triblock copolymers with the longest PDPA blocks (*i.e. z* = 356 and 460) are not considered to be genuine framboidal vesicles.

TEM, DLS and zeta potential data suggest that relatively small, rather ill-defined cationic aggregates are formed below pH 5 by this series of G_58_H_300_D_*z*_ triblock copolymers. In order to provide further morphological insights, SAXS patterns were recorded at pH 3. The low *q* gradient of the SAXS pattern obtained for the G_58_H_350_D_86_ triblock copolymer at this pH tends to zero (see Fig. 5, pH 3, orange data), which is characteristic of spherical micelles.^51^ The spherical micelle model^48^,^52^ provided a satisfactory fit to this SAXS pattern over three orders of magnitude of X-ray scattering intensity. The SAXS-derived mean sphere diameter *D*_s_ (where *D*_s_ = 2*R*_s_ + 4*R*_g_) was calculated to be 33.6 ± 3.2 nm, which is consistent with that reported by DLS (44 nm, see Table 1) given that these two techniques report different moments of the size distribution. However, the SAXS patterns recorded for G_58_H_300_D_164_ and G_58_H_300_D_249_ at pH 3 show a significant upturn in X-ray scattering intensity at low *q* compared to the pattern obtained for G_58_H_300_D_86_ at pH 3. This suggests that scattering objects larger than the spherical micelles have been formed, hence the spherical micelle model alone is not appropriate for data analysis of these two copolymer compositions. TEM analysis (Fig. 2, pH 3) indicates the formation of mass fractals comprising aggregates of weakly-interacting spherical micelles.^53^ Thus, these two SAXS patterns were fitted using a superposition of the spherical micelle model and a truncated power law function representing a mass fractal structure [
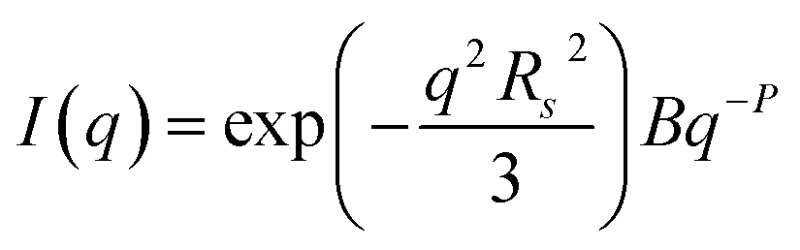
, where *I*(*q*) is the scattered X-ray intensity, *B* is a constant and *P* is an exponent corresponding to the mass fractal dimension; the exponent term associated with *R*_s_ provides a high *q* cut-off for the power component^54^]. This approach gave satisfactory fits to the data over nearly four orders of magnitude of X-ray scattering intensity (see Fig. 5, pH 3, blue and green data). Smaller micelles were formed at pH 3 when increasing the DP of the PDPA block. For example, *D*_s_ was determined to be 30.8 ± 2.8 nm and 26.9 ± 2.6 nm for *z* = 164 and 249, respectively (Table S1†). This is in good agreement with the well-known principles of block copolymer self-assembly: longer stabiliser blocks favour the formation of micelles with lower aggregation numbers.^55^–^57^ Moreover, the Percus–Yevick effective volume fraction (*F*_PY_) increased from 0.08 to 0.12 on increasing *z* from 164 to 249, indicating that the smaller spherical micelles become more aggregated under these conditions. This is consistent with the observed increase in the mass fractal dimensions (*P*) from 1.61 to 1.88, suggesting the formation of denser fractals when *z* = 249. In summary, the precise copolymer morphology obtained at pH 3 is strongly dependent on the DP of the PDPA block. However, we do not have a satisfactory explanation for the formation of these mass fractals (as opposed to non-interacting spherical micelles) at the present time.

A final SAXS experiment utilising a stopped-flow set-up was conducted to examine the precise time scale on which such framboidal vesicles dissociate after addition of acid. This experiment was conducted using a similar framboidal triblock copolymer to those reported in this work. More specifically, a framboidal G_58_H_250_D_184_ triblock copolymer was employed. ^1^H NMR studies indicated that 92% DPA conversion was achieved within 24 h at 70 °C for this particular copolymer synthesis (see Fig. S7a in ESI†). TEM studies confirmed that framboidal vesicles were present at pH 8 (see Fig. S7b in ESI†) and indicated the formation of mass fractals at pH 3 (see Fig. S7c in ESI†). These observations are fully consistent with those discussed above for similar G_58_H_250_D_*z*_ framboidal vesicles.

**Fig. 6:**
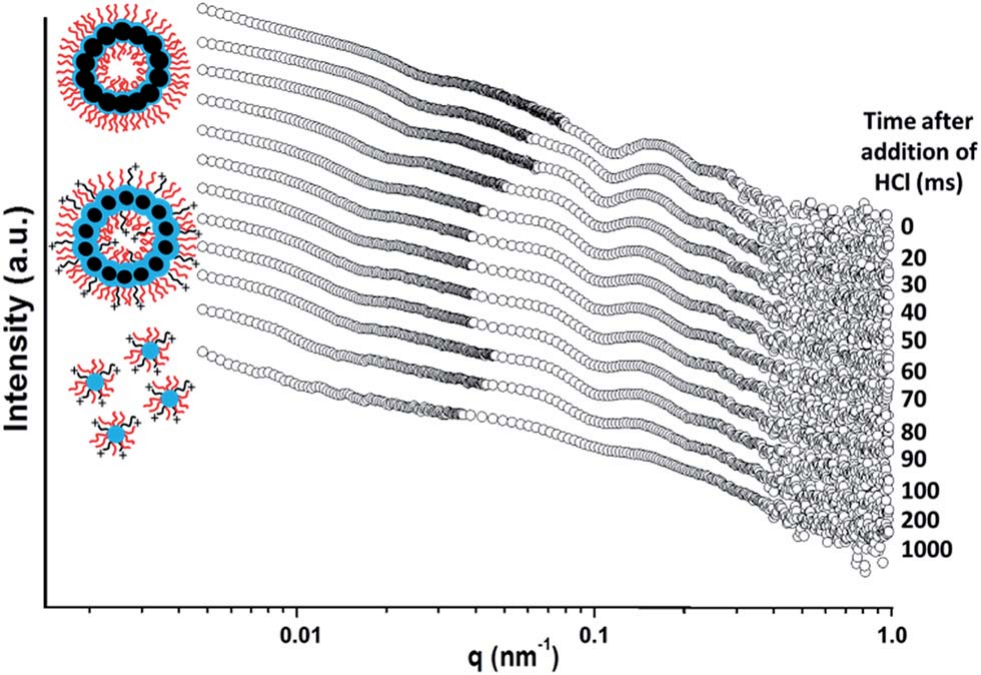
Time-resolved SAXS patterns obtained for a 0.27% w/w aqueous dispersion of G_58_H_250_D_184_ triblock copolymer vesicles after addition of acid using an HCl/DPA molar ratio of 1.50. A scattering pattern was obtained for the G_58_H_250_D_184_ vesicles prior to addition of acid. Scattering patterns were then recorded every 10 ms for 1.0 s. Selected SAXS patterns are shown after various reaction times (for clarity, these patterns are shifted by an arbitrary scaling factor). The framboidal vesicle diameter increases and the vesicle membrane swells as the tertiary amine groups within the PDPA chains become protonated until vesicle disintegration occurs to form mass fractals comprising cationic spherical micelles. [N.B. PGMA = red, PHPMA = blue, PDPA = black].

The initial SAXS pattern obtained for these G_58_H_250_D_184_ vesicles at pH 8 (see the uppermost pattern shown in Fig. 6) resembles that recorded for the G_58_H_300_D_164_ vesicles (see Fig. 5a). To analyse the kinetics of the acid-induced disintegration of these G_58_H_250_D_184_ framboidal vesicles using an HCl/DPA molar ratio of 1.50, a stopped-flow cell was mounted on the synchrotron beamline and SAXS patterns were recorded every 10 ms for 1.0 s. However, the first pattern was excluded, because the dead time for the stopped-flow cell set-up was determined to be 16 ms. Selected SAXS patterns are displayed in Fig. 6.

Detailed analysis of these SAXS patterns has not been attempted, because the complexity of the evolving multi-component system (framboidal vesicles, swollen vesicles and weakly interacting cationic spheres) precludes data fitting to existing scattering models. Nevertheless, the kinetics of vesicle dissociation can be assessed from these curves. The inflection in the first (upper) SAXS pattern at *q* ∼ 0.03 nm^–1^ represents the overall vesicle diameter. This feature shifts to lower *q* over time, indicating the formation of larger vesicles after addition of HCl. Similarly, the local minimum in the first SAXS pattern at *q* ∼ 0.1 nm^–1^ corresponding to the mean membrane thickness (*T*_m_) shifts to lower *q*. This is consistent with substantial membrane swelling due to water ingress as the tertiary amine groups within the PDPA block become protonated and hence hydrophilic. Just 1.0 s after HCl addition, there are no longer any vesicles present within the aqueous dispersion. Instead, rapid vesicle dissociation produces mass fractal aggregates of ill-defined spherical micelles, as indicated by the static measurements discussed above.

## Experimental

### Materials

All reagents were used as received unless otherwise stated. 2-(Diisopropylamino)ethyl methacrylate (DPA), deuterium chloride (DCl; 20% w/w in D_2_O) and 4,4′-azobis-4-cyanopentanoic acid (ACVA) were purchased from Sigma-Aldrich (UK). DPA inhibitor was removed by passing this monomer through an inhibitor removal column. Ethanol, dichloromethane, dimethyl sulfoxide (DMSO) and dimethyl formamide (DMF) were purchased from Fisher Scientific (UK). 2-Cyano-2-propyl dithiobenzoate (CPDB) was purchased from Strem Chemicals (Cambridge, UK). Glycerol monomethacrylate (GMA) was kindly donated by GEO Specialty Chemicals (Hythe, UK) and used without further purification. 2-Hydroxypropyl methacrylate (HPMA) was purchased from Alfa Aesar (UK) and contained 0.07% dimethacrylate impurity, as judged by high performance liquid chromatography (HPLC). Deuterated methanol (CD_3_OD) was purchased from Goss Scientific (UK). Deionised water was obtained using an Elga Elgastat Option 3A water purifier; its pH was approximately 6.2 and its surface tension was around 72.0 mN m^–1^ at 20 °C.

#### RAFT synthesis of PGMA macro-CTA agent in ethanol

A round-bottomed flask was charged with GMA (30.0 g; 187 mmol), CPDB (0.823 g, 2.97 mmol; target DP = 63), ACVA (0.167 g, 0.156 mmol; CPDB/ACVA molar ratio = 5) and ethanol (39.2 g). The sealed reaction vessel was purged with N_2_ for 30 min and placed in a pre-heated oil bath at 70 °C for 135 min. The resulting PGMA macro-CTA (GMA conversion = 80%; *M*_n_ = 15 400 g mol^–1^, *M*_w_/*M*_n_ = 1.13) was purified by precipitation into excess dichloromethane. A mean DP of 58 was calculated for this macro-CTA using ^1^H NMR.

#### Preparation of linear PGMA–PHPMA diblock copolymer *via* RAFT aqueous dispersion polymerisation

PGMA_58_ macro-CTA (2.00 g, 0.210 mmol), HPMA monomer (9.10 g, 63.1 mmol) and deionised water (63.0 g, 15 wt%; purged with N_2_ for 30 min) were weighed into a 100 mL round-bottomed flask and purged with N_2_ for 20 min. ACVA was added (19.6 mg, 0.070 mmol; macro-CTA/ACVA molar ratio = 3.0) and purged with N_2_ for a further 10 min prior to immersion in an oil bath set at 70 °C for 2 h. Finally, the HPMA polymerisation was quenched by cooling to 20 °C with immediate exposure to air.

#### Preparation of PGMA–PHPMA–PDPA triblock copolymer *via* RAFT seeded emulsion polymerisation

PGMA_58_–PHPMA_300_ diblock precursor vesicles (6.00 mL of a 10 wt% dispersion, 11.4 μmol), ACVA (0.637 mg, 2.27 μmol; CTA/ACVA molar ratio = 5.0) and DPA monomer (1.21 g, 5.69 mmol, target DP = 500) were weighed into a 20 mL sample vial and purged with N_2_ for 20 min prior to immersion in an oil bath set at 70 °C for 24 h. Then the DPA polymerisation was quenched by cooling to 20 °C with immediate exposure to air. A series of closely related copolymer syntheses were performed for which the PDPA target DP ranged from 50 to 500. In each case, the pH was determined prior to polymerisation to ensure that the final solution pH was below pH 7.4 for optimal RAFT polymerisation conditions.

### Characterisation

#### ^1^H NMR spectroscopy

All ^1^H NMR spectra were recorded in CD_3_OD or (96/6) CD_3_OD/DCl using a 400 MHz Bruker Avance-400 spectrometer (64 scans averaged per spectrum).

#### Gel permeation chromatography (GPC)

Molecular weights and dispersities were determined using either DMF or THF GPC. The DMF GPC set-up comprised two Polymer Laboratories PL gel 5 μm Mixed C columns operating at 60 °C and connected in series to a Varian 390 LC multi-detector suite (only the refractive index detector was utilised) and a Varian 290 LC pump injection module. The GPC eluent was HPLC-grade DMF containing 10 mM LiBr at a flow rate of 1.0 mL min^–1^. DMSO was used as a flow-rate marker. Calibration was conducted using a series of ten near-monodisperse poly(methyl methacrylate) standards (*M*_n_ = 645–618 000 g mol^–1^). Chromatograms were analysed using Varian Cirrus GPC software (version 3.3) provided by the instrument manufacturer (Agilent). The THF GPC set-up comprised two 5 μm (30 cm) Mixed C columns and a WellChrom K-2301 refractive index detector operating at 950 ± 30 nm. The mobile phase contained 2.0% v/v triethylamine and 0.05% w/v butylhydroxytoluene (BHT) and the flow rate was 1.0 mL min^–1^. A series of ten near-monodisperse poly(methyl methacrylate) standards (*M*_p_ values ranging from 645 to 2 186 000 g mol^–1^) were used for calibration.

#### Dynamic light scattering (DLS)

Intensity–average hydrodynamic diameters were measured at 25 °C using a Malvern Zetasizer NanoZS model ZEN 3600 instrument operating at a fixed scattering angle of 173°. Dilute aqueous dispersions (0.10 wt% copolymer) were analysed using disposable cuvettes and all data were averaged over three consecutive runs to give an *z*-average hydrodynamic diameter *via* the Stokes–Einstein equation.

#### Aqueous electrophoresis

Zeta potentials were determined in the presence of 1 mM KCl using the same model ZEN 3600 Malvern Zetasizer NanoZS instrument equipped with an autotitrator (MPT-2 multipurpose titrator, Malvern Instruments). The solution pH was lowered from 10 to 2 using dilute HCl.

#### Transmission electron microscopy (TEM)

Aqueous copolymer dispersions were diluted at 20 °C to generate 0.10% w/w dispersions. Copper/palladium TEM grids (Agar Scientific, UK) were surface-coated in-house to yield a thin film of amorphous carbon. The grids were then plasma glow-discharged for 30 s to create a hydrophilic surface. One 12 μL droplet of each dispersion was adsorbed onto a freshly glow-discharged grid for 20 s and then blotted with filter paper to remove excess solution. To improve the contrast of the PGMA–PHPMA vesicles, uranyl formate (9 μL of a 0.75% w/v aqueous solution) was placed on each sample-loaded grid for 20 s and then carefully blotted to remove excess stain. To stain the PGMA–PHPMA–PDPA triblock copolymer aggregates, phosphotungstic acid (9 μL of a 1.0% w/v solution) was placed on each sample-loaded grid for 5 s and then carefully blotted to remove excess stain. Each grid was then dried using a vacuum hose. Imaging was performed using a FEI Tecnai Spirit TEM instrument equipped with a Gatan 1kMS600CW CCD camera operating at 120 kV.

#### Small-angle X-ray scattering (SAXS)

SAXS patterns were recorded at the European Synchrotron Radiation Facility (ESRF, Grenoble, France) at stations BM26 and ID02. A monochromatic X-ray radiation (wavelength *λ* = 0.1033 nm and 0.0995 nm, respectively) and 2D SAXS detectors (Pilatus 1M and Rayonix MX-170HS, respectively) were used for these experiments. The SAXS camera length was varied to cover a *q* range from 0.02 nm^–1^ to 1.9 nm^–1^, where 
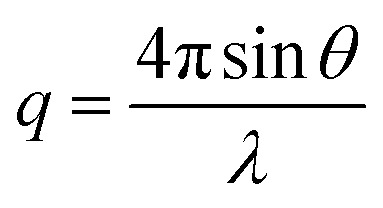
 is the modulus of the scattering vector and *θ* is one-half of the scattering angle. Either polycarbonate or glass capillaries with a diameter of 2.0 mm were used as sample holders. Time-resolved SAXS measurements were performed at ID02 station using a Biologic stopped-flow apparatus. In such experiments, a glass cuvette of 1.4 mm path length was used as the sample holder. Static and time-resolved SAXS measurements were conducted on copolymer dispersions diluted to 1.0% w/w and 0.27% w/w, respectively (with the latter value being the final concentration once aqueous HCl had been added to the aqueous copolymer dispersion in the chamber). Two camera lengths were used to collect the stopped-flow time-resolved SAXS data. The resulting two data sets were subsequently merged at *q* ∼ 0.015 nm^–1^. SAXS patterns recorded at these two camera lengths overlapped well (see Fig. 6 and S8 in the ESI†), indicating excellent reproducibility. X-ray scattering data were reduced (integrated, normalised, background-subtracted) using either standard routines from the ID02 beamline or Nika SAS macros for Igor Pro, and were further analysed by Irena SAS macros for Igor Pro.^58^

## Conclusions

Polymerisation-induced self-assembly has been used to prepare pH-responsive ABC triblock copolymer vesicles *via* RAFT seeded emulsion polymerisation of DPA within PGMA–PHPMA precursor vesicles. Only relatively poor control over the copolymer molecular weight distribution was achieved during growth of the third block. Nevertheless, TEM and SAXS studies indicate that the final PGMA–PHPMA–PDPA vesicles exhibit a distinctive framboidal morphology at around neutral pH, with the globule size correlating with the target DP of the PDPA block. On addition of acid, the tertiary amine groups within this block become protonated and hence hydrophilic. This drives vesicular disintegration: only ill-defined aggregated spheres are observed by post mortem TEM analysis and this dramatic change in copolymer morphology is irreversible. SAXS studies confirm the presence of a fractal-like morphology at pH 3, whose features depend on the DP of the PDPA block. Furthermore, time-resolved SAXS studies performed after addition of acid reveal a substantial increase in vesicle diameter and a significantly thicker vesicle membrane after just 50 ms, with vesicle disintegration being complete within one second.

## Conflicts of interest

There are no conflicts to declare.

## Supplementary Material

Supplementary information
