# Supplementary material for: Synthesis and pH-responsive dissociation of framboidal ABC triblock copolymer vesicles in aqueous solution
Source: Chem Sci. 2017 Dec 13;9(6):1454–63. doi: 10.1039/c7sc04788f (PMC5885938; doi:10.1039/c7sc04788f)
Supplement: Supplementary file 1 [file SC-009-C7SC04788F-s001.pdf]

Electronic Supplementary Material (ESI) for Chemical Science.
This journal is © The Royal Society of Chemistry 2017

Supporting Information for manuscript:

"Synthesis and pH-Responsive Dissociation of Framboidal ABC Triblock Copolymer Vesicles in Aqueous Solution"

C. J. Mable, L. A. Fielding, M. J. Derry, O. O. Mykhaylyk, P. Chambon and S. P. Armes

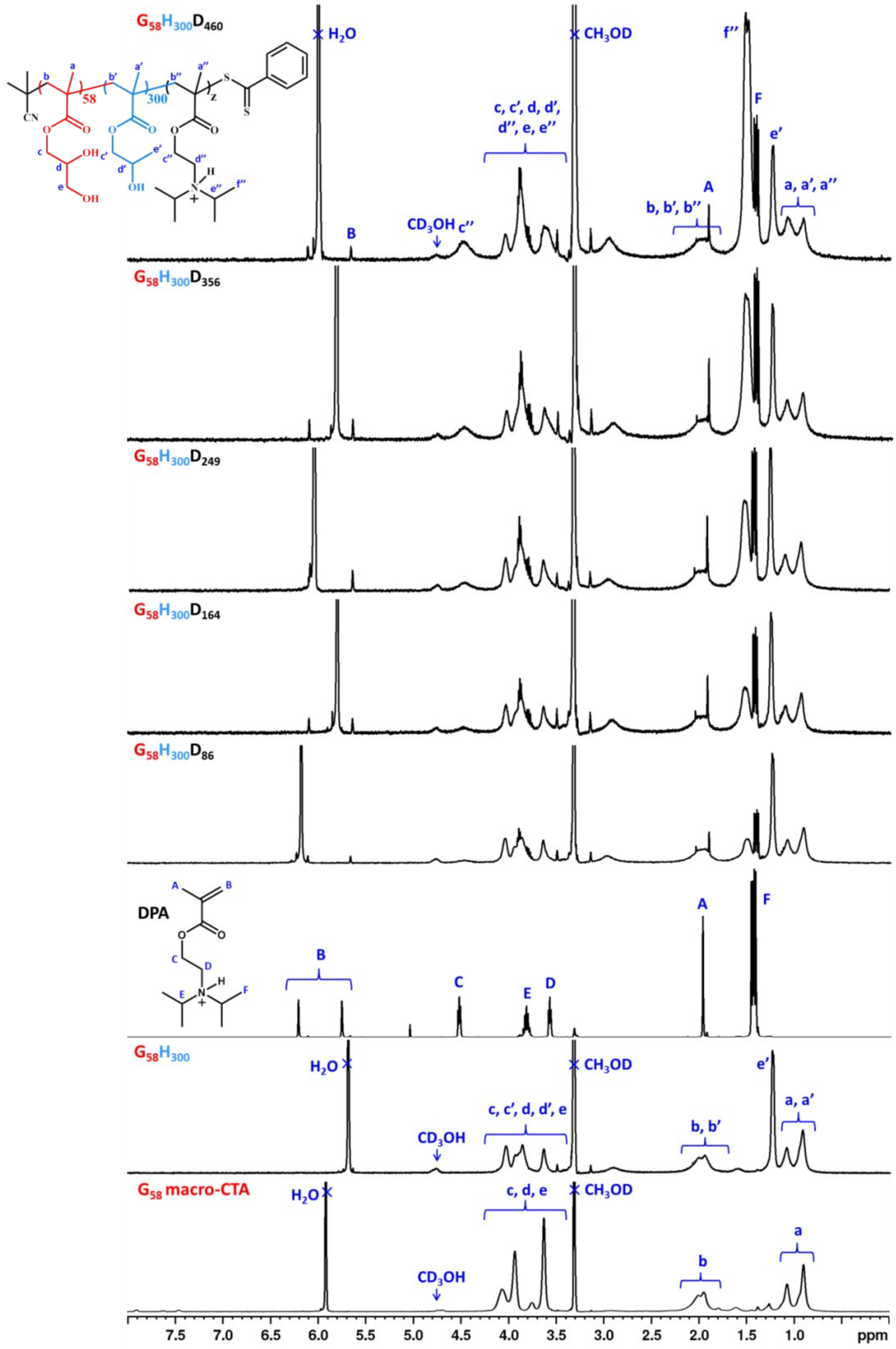

Fig. S1. Assigned ^{1}H NMR spectra in CD_3OD plus 4 % DCl/D_2O (20% w/w DCl) recorded for the G_{58} macro-CTA, $G_{58}H_{300}$ diblock copolymer, DPA monomer and $G_{58}H_{300}D_z$ triblock copolymers (where z = 86, 164, 249, 356 and 460).

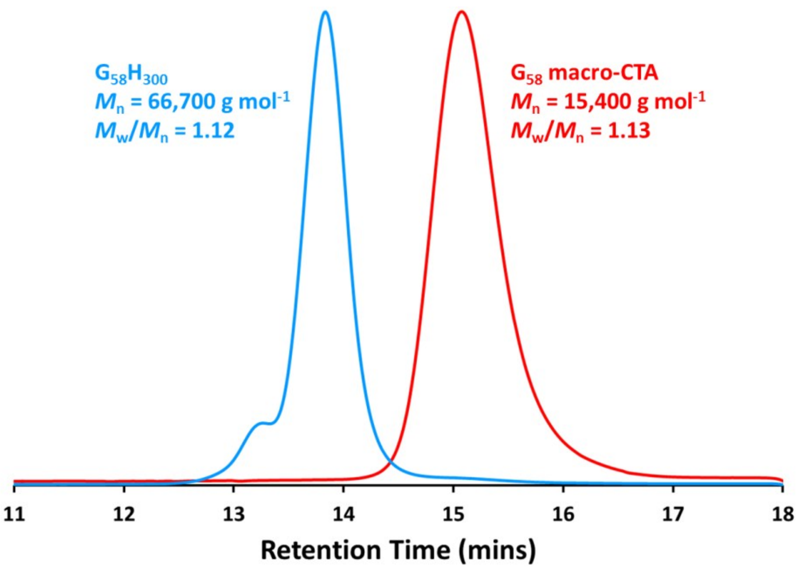

Fig. S2. DMF GPC curves (vs. a series of near-monodisperse PMMA standards) obtained for the G_{58} macro-CTA (red) and the corresponding $G_{58}H_{300}$ diblock copolymer precursor (blue).

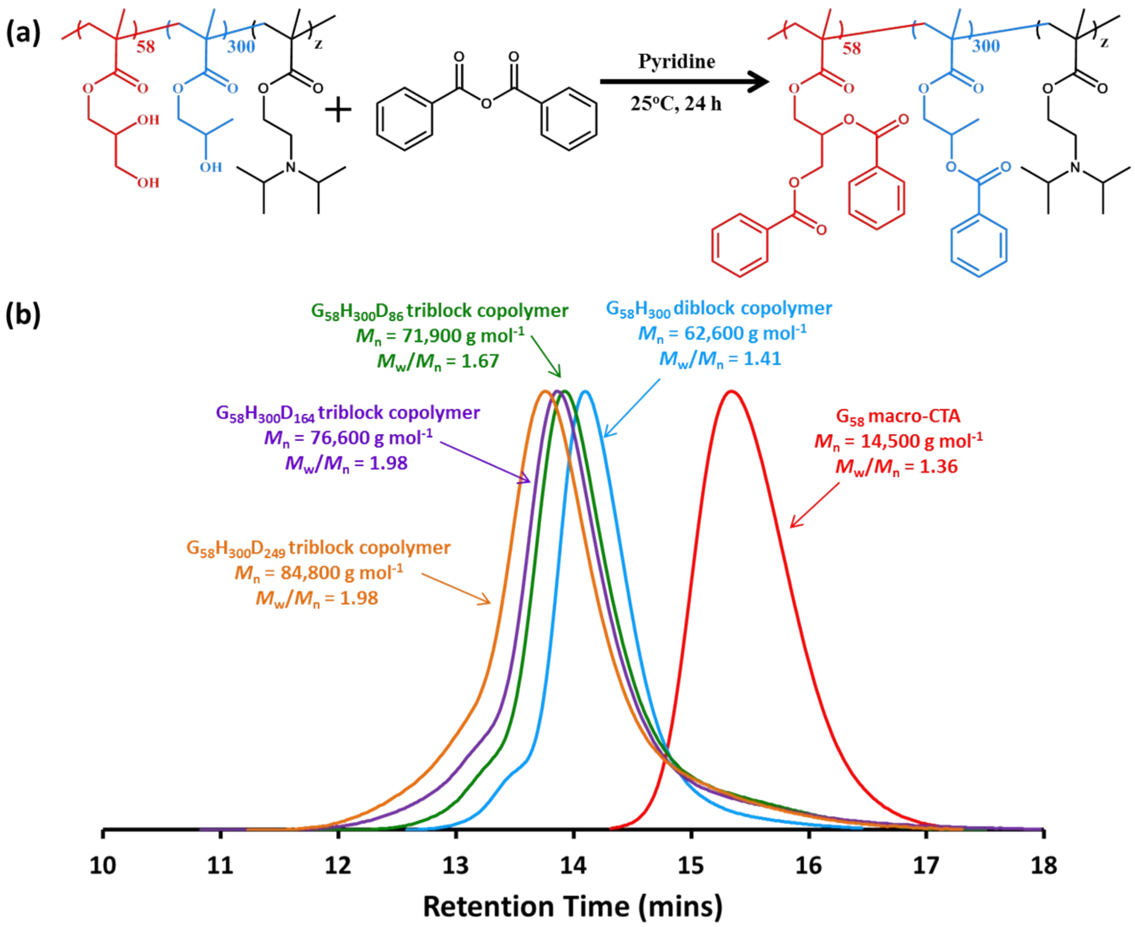

Fig. S3. (a) Reaction scheme for the esterification of GMA and HPMA residues of the triblock copolymers using excess benzoic anhydride. (b) THF GPC curves (vs. a series of near-monodisperse PMMA standards) obtained for the benzoate-modified G_{58} macro-CTA, $G_{58}H_{300}$ diblock copolymer precursor and four $G_{58}H_{300}D_z$ triblock copolymers (where z = 86, 164, 249 or 460).

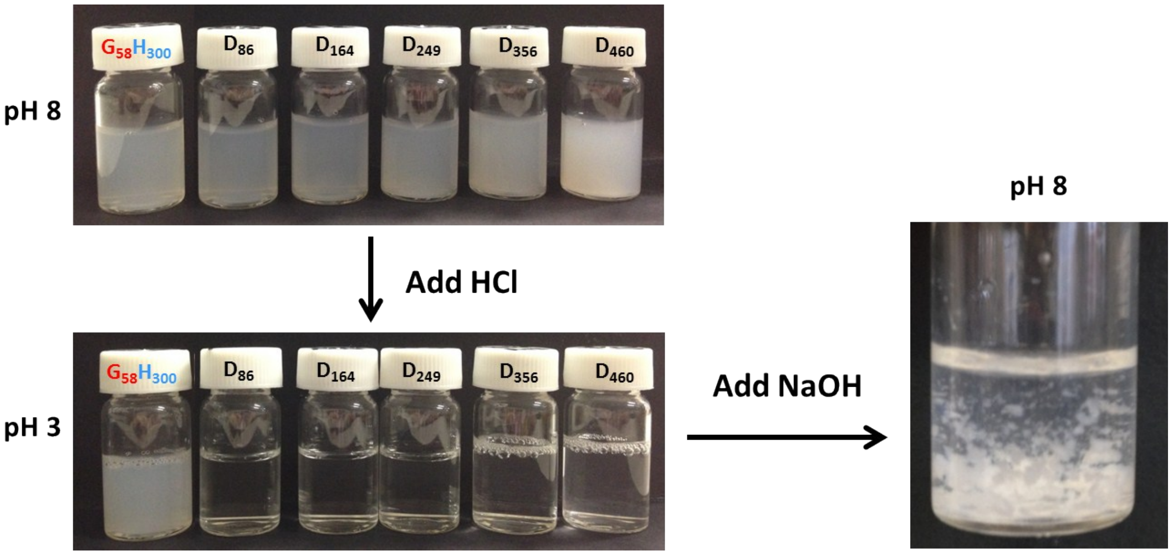

Fig. S4. Digital photographs recorded for $G_{58}H_{300}D_z$ triblock copolymer vesicle dispersions (where z is 86, 164, 249, 356 or 460) at pH 8 and the resulting change in turbidity after switching to pH 3.

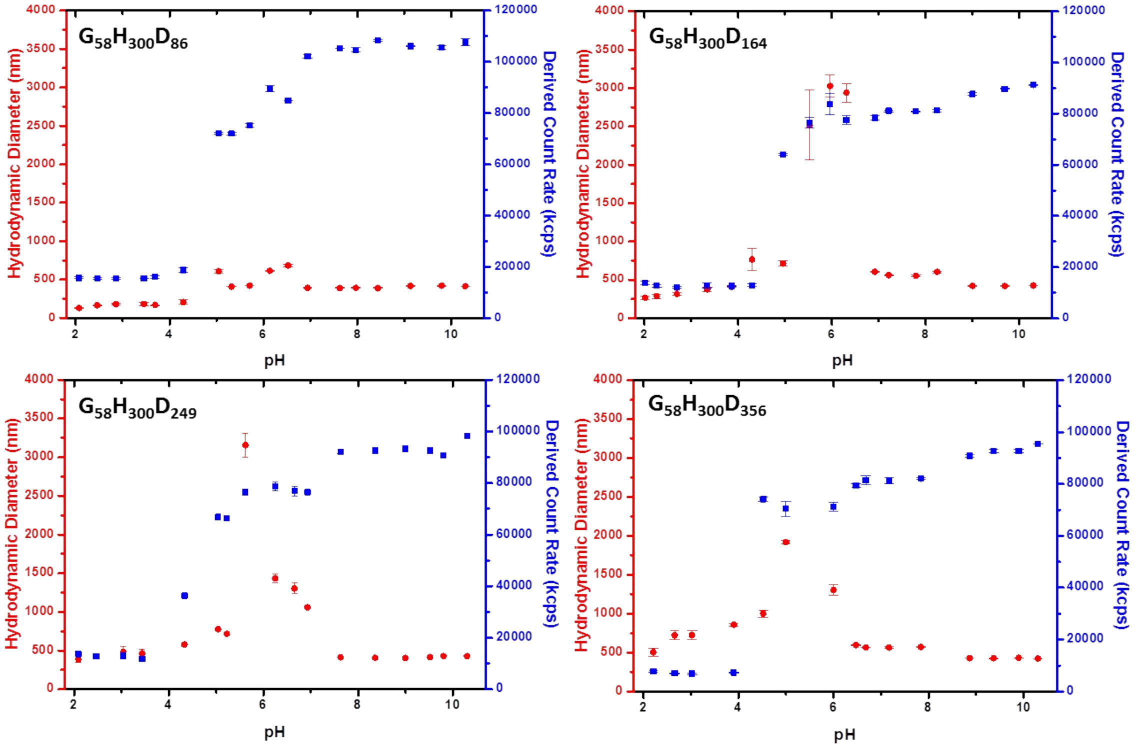

Fig. S5. Variation of the hydrodynamic diameter (red ●) and count rate (blue ■) with solution pH for (top left) $G_{58}H_{300}D_{86}$ triblock copolymer vesicles, (top right) $G_{58}H_{300}D_{164}$ triblock copolymer vesicles, (bottom left) $G_{58}H_{300}D_{249}$ triblock copolymer vesicles and (bottom right) $G_{58}H_{300}D_{356}$ triblock copolymer vesicles.

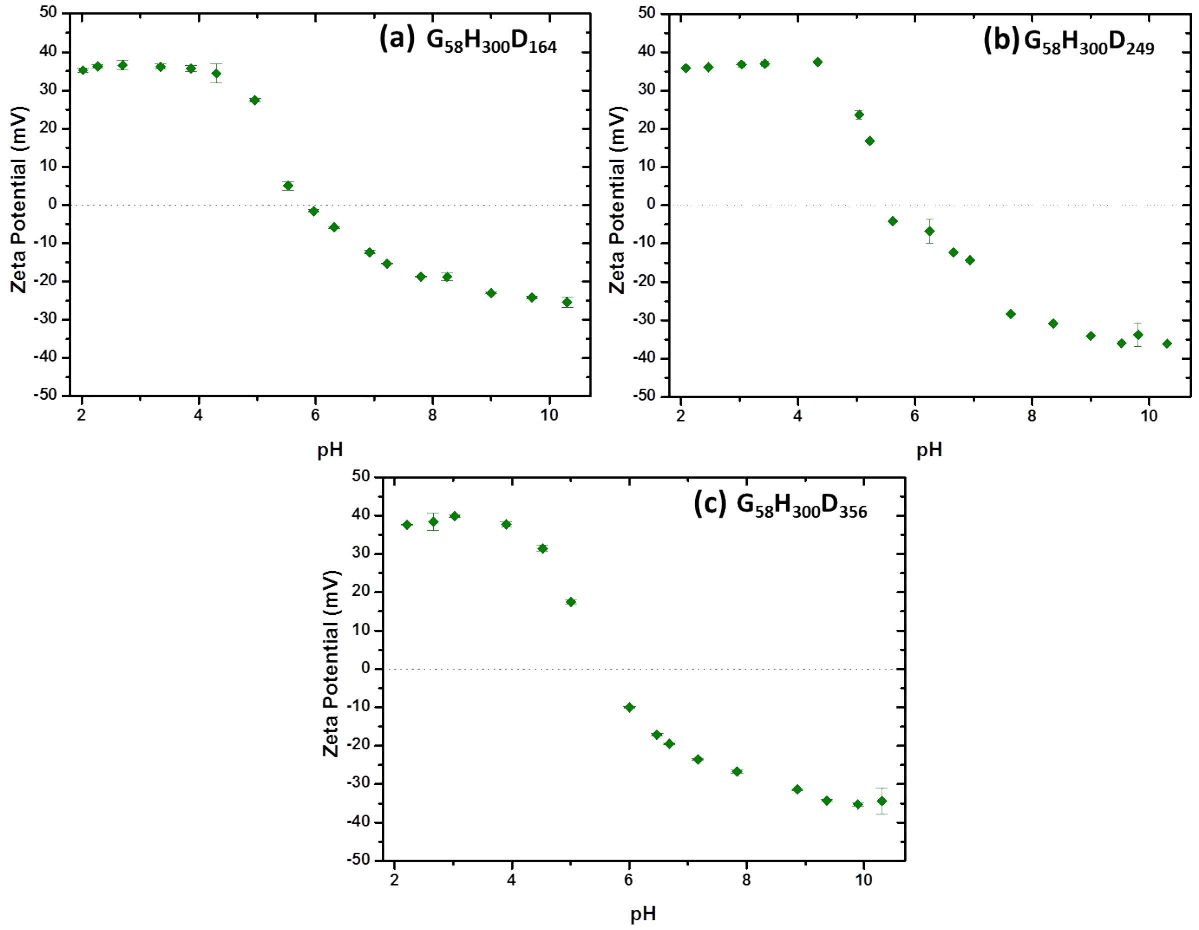

Fig. S6. Variation in zeta potential with solution pH for (a) $G_{58}H_{300}D_{164}$ triblock copolymer vesicles, (b) $G_{58}H_{300}D_{249}$ triblock copolymer vesicles and (c) $G_{58}H_{300}D_{356}$ triblock copolymer vesicles.

Table S1. Structural parameters obtained for a series of $G_{58}H_{300}D_z$ (z = 0 to 249) aqueous copolymer dispersions from SAXS analysis. The volume and scattering length density of the brush/corona block (V_{brush} and ξ_{brush}, respectively) and the core block (V_{core} and ξ_{core}, respectively) were fixed parameters for fitting, based on theoretical calculations. Representative parameters for **population 1** corresponding to vesicles: R_{mc} is the radius from the centre of the vesicle to the centre of the membrane and σ_{Rmc} is the associated standard deviation, T_{mc} is the thickness of the hydrophobic part of the vesicle membrane and σ_{Tmc} is the associated standard deviation, D_{SAXS} is the vesicle diameter: $D_{SAXS} = 2(R_{mc} + \frac{1}{2}T_{mc} + 2R_g)$, where R_g is the radius of gyration of the brush/corona block. Representative parameters for **population 2** corresponding to spherical micelles: R_s is the core radius, σ_{Rs} is the standard deviation of the core radius, R_{PY} is the Percus-Yevick correlation radius of densely-packed spherical micelles (this parameter should be doubled for the inter-particle correlation distance) and F_{PY} is the Percus-Yevick effective volume fraction of the packed micelles. c_2/c_1 is the ratio of the copolymer volume fraction forming spherical micelles (**population 2**) to that forming vesicles (**population 1**). Finally, D is the mass fractal dimensions for the mass fractal model (**population 3**).

Copolymer Composition	pH	V_{brush}[a] /nm^3	V_{core}[b] /nm^3	ξ_{brush}[a] $\times 10^{10}$ /cm^{-2}	ξ_{core}[b] $\times 10^{10}$ /cm^{-2}	Population 1 - Vesicles			c_2/c_1	Population 2 – Spherical Micelles			Population 3 - Mass Fractals
						R_{mc} (σ_{Rmc})[c] /nm	T_{mc} (σ_{Tmc})/ nm	D_{SAXS}[c]/ nm		R_s (σ_{Rs})/ nm	R_{PY} /nm	F_{PY} /nm	D
$G_{58}H_{300}$	8.0 and 3.0	11.8	59.4	11.94	11.11	178 (40)	16.8 (1.8)	383					
$G_{58}H_{300}D_{86}$	8.0	11.8	88.3	11.94	10.71	178 (49)	24.9 (1.3)	391	0.273	8.5 (1.2)	11.9	0.34	
	3.0	40.7	59.4	10.48	11.11					11.9 (1.6)	12.0	0.05	
$G_{58}H_{300}D_{164}$	8.0	11.8	114	11.94	10.52	178 (50)	36.3 (1.5)	402	0.701	13.7 (2.6)	15.3	0.45	
	3.0	66.9	59.4	10.25	11.11					10.5 (1.4)	10.8	0.08	1.61
$G_{58}H_{300}D_{249}$	8.0	11.8	143	11.94	10.40	178 (45)	46.4 (1.3)	412	0.997	17.0 (4.1)	20.7	0.53	
	3.0	95.5	59.4	10.14	11.11					8.5 (1.3)	10.0	0.12	1.88

[a] At pH 8.0, $V_{brush} = \frac{M_{n,PGMA}}{N_A \rho_{PGMA}}$ and $\xi_{brush} = \xi_{PGMA}$. At pH 3.0, $V_{brush} = \frac{M_{n,PGMA}}{N_A \rho_{PGMA}} + \frac{M_{n,PDPA}}{N_A \rho_{PDPA}}$ and $\xi_{brush} = \varphi_{PGMA}\xi_{PGMA} + (1 - \varphi_{PGMA})\xi_{PDPA}$ where φ_{PGMA} is the volume fraction of PGMA in the corona.

[b] At pH 8.0, $V_{core} = \frac{M_{n,PHPMA}}{N_A \rho_{PHPMA}} + \frac{M_{n,PDPA}}{N_A \rho_{PDPA}}$ and $\xi_{core} = \varphi_{PHPMA}\xi_{PHPMA} + (1 - \varphi_{PHPMA})\xi_{PDPA}$ where φ_{PHPMA} is the volume fraction of PHPMA in the core-forming block. At pH 3.0, $V_{core} = \frac{M_{n,PHPMA}}{N_A \rho_{PHPMA}}$ and $\xi_{core} = \xi_{PHPMA}$.

[c]These data are considered less reliable because the camera length used to obtain the SAXS data was not long enough to give accurate overall vesicle diameters.

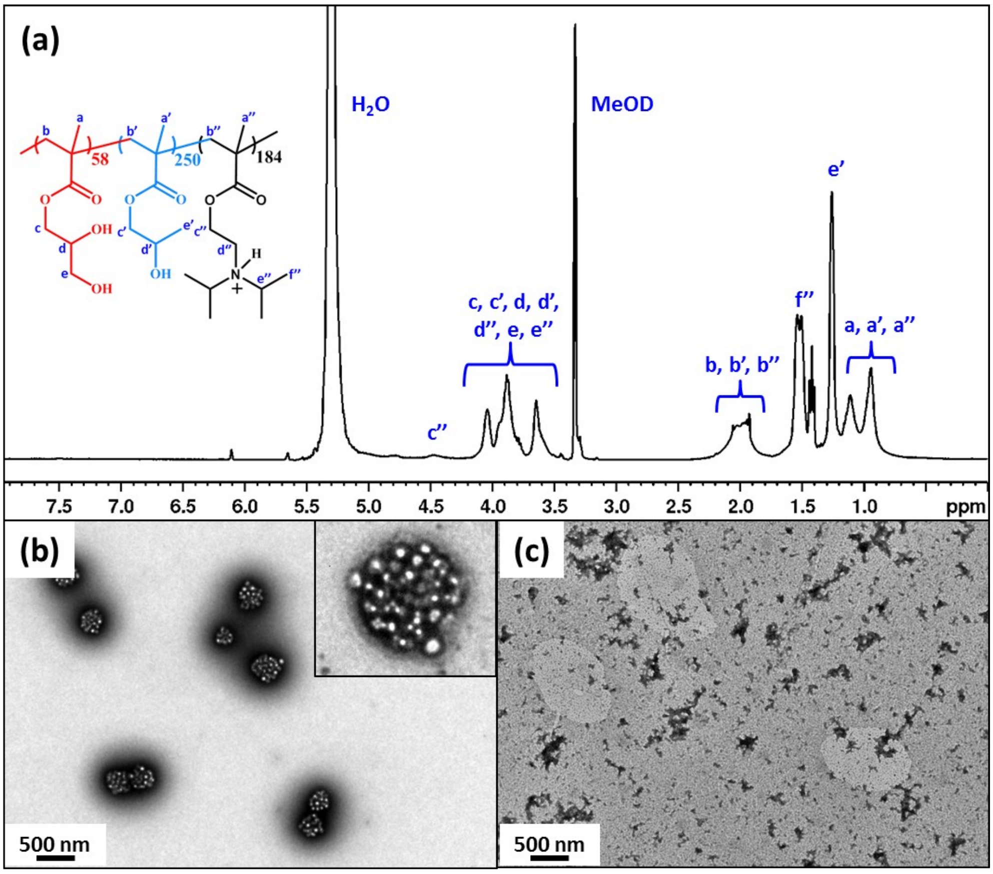

Fig. S7. (a) Assigned ^{1}H NMR spectrum of the $G_{58}H_{250}D_{184}$ triblock copolymer recorded in CD_3OD plus 4 % DCl/D_2O (20% w/w DCl). Representative TEM images obtained for (b) $G_{58}H_{250}D_{184}$ framboidal triblock vesicles at pH 8 and (c) fractal aggregates of cationic spheres formed by the same copolymer at pH 3.

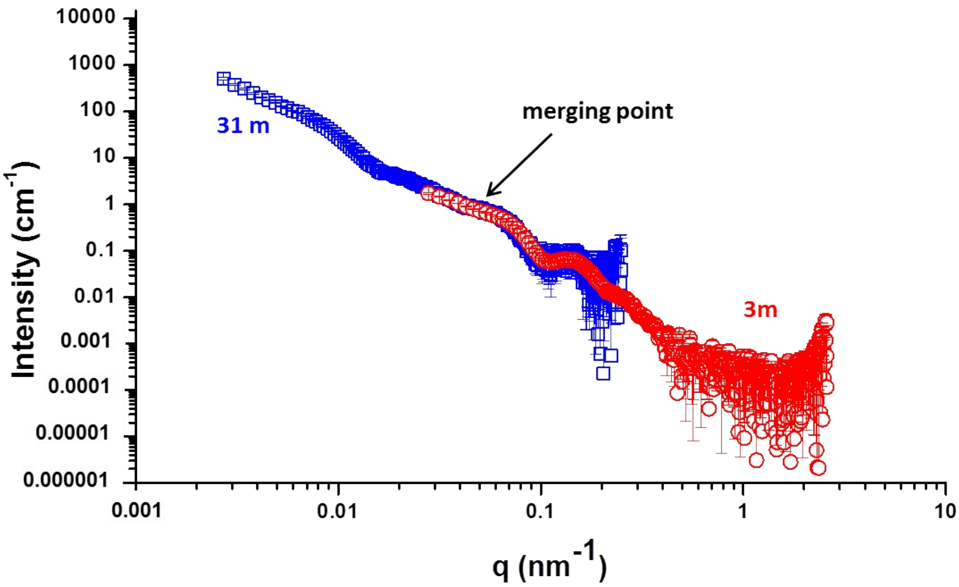

Fig. S8. Two SAXS patterns recorded for $G_{58}H_{250}D_{184}$ framboidal vesicles 100 ms after HCl addition. These patterns were obtained from two identical experiments run using a camera length of 3 m (red data) or 31 m (blue data). The overlap between the two data sets indicates excellent data reproducibility.

References

1. J. Ilavsky and P. R. Jemian, *J. Appl. Crystallogr.*, **2009**, *42*, 347-353
